# Sarcopenia, sarcopenic obesity, and arterial stiffness among older adults

**DOI:** 10.3389/fcvm.2024.1272854

**Published:** 2024-02-08

**Authors:** Francesco Fantin, Anna Giani, Gisella Manzato, Annachiara Zampieri, Gabriele Comellato, Silvia Urbani, Elena Zoico, Gloria Mazzali, Mauro Zamboni

**Affiliations:** ^1^ Section of Geriatric Medicine, Department of Medicine, University of Verona, Verona, Italy; ^2^ Section of Geriatric Medicine, Department of Surgery, Dentistry, Pediatric and Gynecology, University of Verona, Verona, Italy

**Keywords:** arterial stiffness, sarcopenia, obesity, sarcopenic obesity, cardio-ankle vascular index, pulse wave velocity

## Abstract

**Background:**

Aging is associated with a higher prevalence of sarcopenia, sarcopenic obesity (SO), and increased arterial stiffening, with possible detrimental effects on morbidity and mortality. The aim of this study was to assess the relationships between sarcopenia, SO, and different indexes of arterial stiffness in older adults.

**Methods:**

A total of 77 hospitalized patients (mean age 78.68 ± 9.65 years) were evaluated, obtaining anthropometric variables, biochemical samples, handgrip test, and body composition assessment. Arterial stiffness was evaluated by measuring both carotid-femoral pulse wave velocity (cfPWV), a proxy for central stiffness, and cardio-ankle vascular index (CAVI), as well as considering peripheral arteries. The population was sorted into four subgroups: obese, sarcopenic, SO, and controls.

**Results:**

The highest CAVI (11.31 ± 2.58) was found in sarcopenic patients. SO had the highest value of cfPWV (15.18 ± 8.44 m/s), even after adjustment for significant covariates. In multiple regressions, SO diagnosis resulted as a significant predictor of cfPWV (*p* = 0.03, *R*^2 ^= 0.20), and sarcopenia diagnosis resulted as a predictor of CAVI (*p* = 0.042, *R*^2 ^= 0.12).

**Conclusions:**

In conclusion, a positive correlation is found between sarcopenia, SO, and arterial stiffness among older subjects. In particular, greater central arterial stiffness is associated with SO, outlining a remarkable effect on the cardiovascular risk profile.

## Introduction

In the past years, two epidemics, aging and obesity, have spread worldwide, drawing greater attention to several conditions that typically affect morbidity, mortality, and disability. The subpopulation of adults aged 65 years and over already accounts for 13% of the world population, and it is expected to steeply increase to over 2 billion people in 2050. Both in Europe (1) and in the US (2), the prevalence of obesity increased following the aging population trend, with around 16% (in Europe) and 35% (in the US) of adults over 65 years considered obese.

In this scenario, obesity (3) and sarcopenia (4), along with cardiovascular disorders, are frequent findings among older adults, and they both contribute to the burden of functional impotence and disability (5).

Recently, the concomitant presence of sarcopenia and obesity has been characterized in detail and defined as sarcopenic obesity (SO) (6), a unique clinical and functional entity that relies on the combined effect of these two conditions, differing from obesity or sarcopenia alone (7–10).

Previous evidence outlined remarkable associations between sarcopenia, SO, morbidity, and mortality (6, 11), yet there is a relative lack of knowledge regarding the possible relationship connecting sarcopenia, SO, and cardiovascular risk. Sarcopenia has been proven to relate to cardiovascular diseases (12). Several factors that are involved in the pathogenesis of SO, such as excessive caloric intake, physical inactivity, low-grade inflammation, insulin resistance, and hormonal changes (10), are well-known cardiovascular risk factors (13); however, a solid proof of the connection between SO and cardiovascular risk is still missing.

Arterial stiffness represents an intermediate endpoint in physiological aging and overt pathological conditions, pinpointing vascular aging prior to the plain onset of cardiovascular morbidity. Arterial stiffening is part of a complex network of inflammatory and atherogenic pathways (14) contributing to reduced arterial wall elasticity and compliance (15). Different techniques may be applied to explore different features of vascular stiffening, and the concomitant evaluation of both peripheral and central arterial segments, with separate tools, provides a comprehensive understanding of the regionality of the stiffening process. In fact, tonometric evaluation of carotid-femoral Pulse Wave Velocity (cfPWV) describes central arterial segments, and it is deemed a reliable predictor of mortality risk in different subsets of patients (16); however, its trend in sarcopenia and SO has scarcely been explored. On the other hand, the cardio-ankle vascular index (CAVI), which is used to evaluate arterial stiffness from a larger proportion of the arterial tree, is less dependent on blood pressure at the time of measurement (17, 18). More evidence is provided using CAVI, which allows the extrapolation of brachial-ankle PWV (baPWV) (19, 20), observing higher values of CAVI and baPWV in subjects with sarcopenia than in controls and demonstrating that sarcopenia can independently affect arterial stiffness (19). Consolidated knowledge identified increased arterial stiffness in obese (mostly visceral obesity) subjects (21).

Nevertheless, the relationship between arterial stiffness and SO is yet to be entirely described, and it may be hypothesized that in an SO setting, the synergistic effect of sarcopenia and obesity may also play a greater role in the cardiovascular risk profile.

Thus, the aim of this study was to assess the relationships between sarcopenia, SO, and different indexes of arterial stiffness in a group of older adults.

## Materials and methods

### Study population

A total of 77 Caucasian older adults, hospitalized at the Geriatric Division of Verona University Hospital, were enrolled. Each subject underwent a comprehensive clinical evaluation, recording medical history (with particular attention to cardio-metabolic disorders) and performing a whole physical examination.

### Anthropometry

Body weight was measured with the subject barefoot and wearing light indoor clothing (Salus scale, Milan, Italy). Height was measured using a stadiometer, with an approximation of 0.5 cm (Salus Stadiometer Milan, Italy). Body mass index (BMI) was calculated as the ratio between weight and height squared (kg/m^2^). Waist circumference (WC) was also obtained with a measuring tape at the narrowest circumference of the abdomen: Men with a WC larger than 102 cm and women with a WC larger than 88 cm were classified as obese (22).

### Sarcopenia and sarcopenic obesity diagnosis

Sarcopenia was assessed according to the latest algorithm suggested by international guidelines (23). To find cases, clinical symptoms of sarcopenia were first assessed; to assess for evidence of sarcopenia, the grip strength test was systematically performed; to confirm sarcopenia diagnosis, low muscle quantity and quality were assessed by Bioimpedance analysis (BIA). Subjects with grip strength and muscle mass lower than the suggested cut-off were considered sarcopenic.

Sarcopenic obesity was diagnosed according to the latest consensus guidelines (6), in particular, as screening measures, BMI, WC, and clinical symptoms of sarcopenia have been investigated; in the “diagnosis step”, muscle strength was measured as handgrip strength, and body composition was assessed by BIA.

### Muscle strength

Handgrip test was performed as a proxy for muscle strength, considering the strength of the flexor muscle of the dominant hand, by a portable dynamometer (Jamar Handheld Dynamometer, Sammons Preston Rolyan, IL, USA). Each subject performed three measurements and the highest value was registered; as suggested by previous evidence, and upon the study population characteristics, the normality threshold values were chosen at 30 kg for men and 20 kg for women (24).

### Body composition

BIA resistance was used to evaluate muscle mass, using a bioimpedance analyser (Human IM Touch, Dietosystem DS Medica s.r.l, Milan, Italy). Whole body BIA measurements were obtained relying on the tetrapolar method, placing the electrodes at the right wrist and ankle with the subject in a supine position, laying on a non-conducting surface, as per the manufacturer's recommendation. At the time of the examination, each patient had fasted for at least 4 h, had an empty bladder, and did not wear any metal object. None of the patients had a cardiac electronic pacemaker. Janssen equation (25) was applied to calculate muscle mass:

$$\begin{array}{rl} \mathrm{Muscle}\;\mathrm{mass}\;(\mathrm{kg})= & \;\;[\left(\mathrm{Heigh}t^{2}|R\times 0.401)+(\mathrm{sex}\times 3.825\right)\\ & +(\mathrm{age}\times -0.071)]+5.102 \end{array}$$

where height is measured in cm; R stands for BIA-resistance (Ohm); sex is represented by factor 1 for men and 0 for women; age is expressed in years. Skeletal muscle mass was divided by the square of the height [muscle mass (kg)/height (m)^2^] to obtain Skeletal Muscle Index (SMI) (26). Fat mass was consequently derived.

Combining available data, sarcopenia was defined in men with SMI ≤ 10.75 kg/m² plus a handgrip <30 Kg and in women with SMI ≤ 6,75 kg/m² plus a handgrip <20 Kg (26); obese sarcopenic individuals had both criteria for obesity and sarcopenia.

### Blood pressure and arterial stiffness

Blood pressure was measured three times, with the patient laying in a supine position, after 10 min of rest, using an aneroid sphygmomanometer (Heine Optothecnik, Gilching, Germany) in the subject's non-dominant arm. The mean value of the three evaluations was considered. Systolic (SBP) and diastolic (DBP) blood pressure levels were collected. Pulse Pressure (PP), which is an independent risk factor for cardiovascular morbidity (27), was obtained. Mean arterial pressure (MAP) was then derived, following the formula:

MAP(mmHg)=DBP+1/3(PP).

Arterial applanation tonometry was performed at the common carotid artery site, using a portable device, PulsePen (Diatecne, Milan, Italy), based on its software WPulsePen 2.0.1. By means of arterial tonometry, Pulse Wave Analysis was performed, and central (cfPWV) velocities were collected, as we previously described (28). A single probe was used, maintaining a double lead ECG recording, as per the manufacturer's protocol (29).

Furthermore, Cardio-Ankle Vascular Index was obtained for each patient, using Vasera VS-1500 (Fukudadenshi Company, Ltd, Tokyo, Japan) (30). As per the manufacturer's recommendation, BP cuffs were placed simultaneously on the four limbs and inflated two by two (right and left side). At the same time, ECG was obtained by two electrodes, and a microphone was placed on the sternum (second rib space) to obtain phonocardiography. CAVI was automatically calculated on the basis of the Bramwell-Hill Formula (31, 32), which relies on PWV, by the following equation:

$$\mathrm{CAVI}=a*\left(\ln\frac{P_{s}}{P_{d}}*\;\frac{PWV^{2}*2\rho }{P_{s}-\;P_{d}}\right)+b$$

a and b are constants, ρ is considered the blood density, P_s_ stands for SBP, and P_d_ stands for DBP.

### Biochemical parameters

Venous blood samples for all metabolic assessments were obtained after the subjects fasted overnight. Plasma glucose was measured with a glucose analyzer (Roche Cobas 8,000, Monza, Italy). Cholesterol and triacylglycerol concentrations were determined with the spectrophotometric method (Roche Cobas 8,000, Monza, Italy). High-density-lipoprotein (HDL) cholesterol was measured using the method of Warnick and Albers. LDL cholesterol was calculated using the Friedwald formula. Creatinine was measured by a modular analyzer (Roche Cobas 8,000, Monza, Italy); the estimated Glomerular Filtration Rate (eGFR) was calculated by the Cockroft-Gault formula.

### Statistical analysis

Results are shown as mean ± standard deviation (SD). Variables not normally distributed were log-transformed before analysis. Independent Samples *t*-tests were used to compare baseline characteristics of male and female populations and the Chi-Square test was used to compare the prevalence of the main diseases between male and female populations.

Pearson's correlations were used to test the relationship between the variables.

The study population was subdivided according to obesity and/or sarcopenia diagnoses; four subgroups were outlined: group 1 was a control group (patients had neither sarcopenia nor obesity, *n* = 8), group 2 was obese patients (*n* = 31), group 3 was sarcopenic patients (*n* = 21), and group 4 was SO patients (*n* = 12).

The analysis of the variance (ANOVA) and covariance (ANCOVA) were used to compare the main variables of the four groups. A *post hoc* analysis was used to evaluate the differences between the four groups. ANCOVA models were adjusted for age, sex, MAP, and LDL cholesterol; covariates were chosen upon Pearson's correlation significance.

Two separate backward regression models were built to evaluate the joint effect of independent variables on cfPWV and on CAVI; independent variables were chosen upon pathophysiological and clinical significance. In the regression models, sarcopenia and sarcopenic obesity were considered diagnostic categories, considering patients with sarcopenia alone (vs. all the other subgroups) and with SO (vs. all the other subgroups). When considering cfPWV as a dependent variable, MAP, heart rate, glycemia, and SO diagnoses were taken as independent variables. When considering CAVI as a dependent variable, MAP, heart rate, glycemia, and sarcopenia diagnoses were considered independent variables; in a further model, cfPWV was added to the other variables in order to relieve the burden of central aortic stiffness.

A *p*-value lower than 0.05 was considered significant. All analyses were performed using the SPSS statistical program (version 20.0 for Windows) and R version 4.2.2 (2022, The R Foundation for Statistical Computing).

The study was approved by the Ethical Committee of the University of Verona.

## Results

The main characteristics of the study population are listed in Table 1. Seventy-seven subjects (mean age 78.68 ± 9.65 years) were evaluated, 56% of whom (*n* = 43) were women.

**Table 1:** Characteristics of the study population, comparing male and female subjects.

	Total (*n* = 77)	Men (*n* = 34)	Women (*n* = 43)	*p*
Age (years)	78.68 ± 9.65	79.97 ± 8.63	77.65 ± 10.37	0.298
Weight (kg)	71.55 ± 18.71	71.81 ± 16.02	71.34 ± 20.78	0.915
BMI (Kg/m²)	26.34 ± 6.19	24.71 ± 5.97	27.62 ± 6.11	0.039
Waist circumference (cm)	97.78 ± 15.63	97.45 ± 16.37	98.25 ± 15.11	0.83
Fat mass (kg)	23.2 ± 12.1	18.93 ± 11.63	26.65 ± 11.46	0.005
Muscle Mass (kg)	46.36 ± 8.85	51.32 ± 8.48	42.35 ± 6.97	0.000
SMI (Kg/m²)	8.35 ± 2.38	8.47 ± 1.66	8.25 ± 2.86	0.697
Glycemia (mg/dl)	98.94 ± 29.11	104.62 ± 35.63	94.44 ± 22.11	0.151
Total cholesterol (mg/dl)	155.99 ± 49.61	140.21 ± 46.05	168.38 ± 49.29	0.014
LDL cholesterol (mg/dl)	85.32 ± 42.25	72.05 ± 38.49	95.74 ± 42.57	0.015
HDL cholesterol (mg/dl)	43.42 ± 18.24	42.88 ± 19.25	43.86 ± 17.6	0.819
Triglycerides (mg/dl)	139.42 ± 72.77	129.18 ± 73.11	147.71 ± 72.29	0.272
Creatinine (umol/L)	89.54 ± 51.08	97.68 ± 55.87	83.12 ± 46.62	0.216
Creatinine clearance (ml/min)	63.49 ± 26.80	99.48 ± 58.19	83.53 ± 47.70	0.801
SBP (mmHg)	130.56 ± 18.28	124.35 ± 15.79	135.47 ± 18.79	0.007
DBP (mmHg)	73.4 ± 11.1	72.03 ± 10.66	74.49 ± 11.44	0.338
PP (mmHg)	57.12 ± 13.72	51.94 ± 12.02	61.21 ± 13.71	0.003
MAP (mmHg)	92.45 ± 12.39	89.47 ± 11.34	94.81 ± 12.8	0.060
HR (bpm)	73.51 ± 9.78	73.03 ± 9.57	74.23 ± 9.96	0.614
cfPWV (m/s)	11.92 ± 4.72	11.2 ± 3.43	12.5 ± 5.52	0.236
CAVI	10.28 ± 2.39	10.68 ± 2.63	9.92 ± 2.12	0.179

BMI, body mass index; SMI, skeletal mass index; LDL, low-density lipoprotein; HDL, high-density lipoprotein; SBP, systolic blood pressure; DBP, diastolic blood pressure; PP, pulse pressure; MAP, mean arterial pressure; HR, heart rate; cfPWV, carotid-femoral pulse wave velocity; CAVI, cardio ankle vascular index.

Variables are displayed as mean ± standard deviation.

When comparing female and male populations, the first had a significantly higher BMI (27.62 ± 6.11 kg/m² vs. 24.70 ± 5.97 kg/m² *p* < 0.05) and significantly higher total cholesterol and LDL cholesterol values (168.38 ± 49.29 vs. 140.21 ± 46.05 mg/dl; *p* < 0.05 and 95.74 ± 42.57 vs. 72.05 ± 39.49 mg/dl; *p* < 0.05, respectively). Conversely, male subjects had significantly higher fat-free mass values (51.32 ± 8.48 kg vs. 42.35 ± 6.97; *p* < 0.001).

The prevalence of obesity was 23.5% in men and 46.5% in women (*p* < 0.05); a higher prevalence of sarcopenia was described in the male population (41.2% vs. 16.3% in the female population, *p* < 0.05). The prevalence of SO was 17.6% among men and 13.9% among women (not significant) (data not shown in table).

In the overall cohort, a borderline positive correlation was detected between cfPWV and age (*r* = 0.21; *p* = 0.06) and between cfPWV and fasting glucose (*r* = 0.22; *p* = 0.06). Moreover, CAVI was positively correlated with age (*r* = 0.54; *p* < 0.001) and negatively with SMI (*r* = −0.30; *p* = 0.01) (data not shown in table).

### Subgroups comparison

When comparing the four body composition phenotypes, a higher prevalence of men was described only in the sarcopenic subgroup (70%), whereas men were 38% of controls, 30% of obese, and 50% of SO patients (*p* = 0.056). No significant difference was detected with respect to smoking habit (*p* = 0.3).

Table 2 depicts the main metabolic and hemodynamic variables when comparing the four groups.

**Table 2:** Characteristics of population subgroups in the study.

	Controls (*n* = 13)	Obese (*n* = 31)	Sarcopenic (*n* = 21)	Sarcopenic-obese (*n* = 12)
Age (years)	75.92 ± 4.44*	72.96 ± 9.35^+¥^	85.33 ± 7.14*^+^	80.58 ± 9.96^¥^
Glycemia (mg/dl)	101.31 ± 27.81	96.54 ± 13.95	97.57 ± 43.08	104.75 ± 31.99
Total cholesterol (mg/dl)	160.92 ± 51.64	178.65 ± 53.62*	130.76 ± 33.46*	158.75 ± 38.43
LDL cholesterol (mg/dl)	93.22 ± 36.58*	103.58 ± 47.09^+^	63.52 ± 32.88*^+^	83.83 ± 32.7
HDL cholesterol (mg/dl)	43.69 ± 13.51	48.44 ± 18.21*	37.76 ± 19.9*	47.08 ± 17.22
Triglycerides (mg/dl)	119.92 ± 72.6	138.63 ± 59.93	149.76 ± 90.83	139.75 ± 70.65
Creatinine (umol/L)	80.25 ± 13.36	96.31 ± 64.57	89.43 ± 58.19	82.5 ± 33.24
SBP (mmHg)	133 ± 27.92	135 ± 13.44*	120.95 ± 17.33*	132.83 ± 13.33
DBP (mmHg)	79.15 ± 13.91*	76.25 ± 8.18^+^	65.33 ± 9.33*^+¥^	75.25 ± 10.19^¥^
PP (mmHg)	53.62 ± 19.23	58.75 ± 11.2	55.62 ± 14.1	57.58 ± 13.27
MAP (mmHg)	97.1 ± 17.82*	95.83 ± 8.77^+^	83.87 ± 10.67*^+¥^	94.44 ± 9.45^¥^
HR (bpm)	73.85 ± 7.59	71.52 ± 7.72	74.76 ± 13.74	77.33 ± 7.96
CAVI (U)	9.55 ± 1.77*	9.35 ± 1.97^+^	11.31 ± 2.58*^+^	10.77 ± 2.72
cfPWV (m/s)	10.4 ± 2.93*	11.12 ± 3.88^+^	11.89 ± 3.13^¥^	15.18 ± 8.44*^+¥^
Waist Circumference (cm)	85.81 ± 6.19*	110.30 ± 11.33*^+^	81.12 ± 9.31^+¥^	107.17 ± 8.43*^¥^
Fat Mass (Kg)	16.98 ± 8.47*	31.06 ± 10.26*^+^	11.29 ± 5.75^+¥^	29.46 ± 7.40*^¥^

LDL, low-density lipoprotein; HDL, high-density lipoprotein; SBP, systolic blood pressure; DBP, diastolic blood pressure; PP, pulse pressure; MAP, mean arterial pressure; HR, heart rate; cfPWV, carotid-femoral pulse wave velocity; CAVI, cardio ankle vascular index.

Groups that share the same apex symbol (*, ^+^, or ^¥^) for a given variable significantly differ (*p* < 0.05).

Noteworthily, sarcopenic patients were significantly older than the control group (*p* < 0.01) and older than obese subjects (*p* < 0.001).

Total and LDL cholesterol levels were significantly lower in sarcopenic patients compared to obese patients (*p* < 0.01), yet no significant difference was detected when comparing SO and sarcopenic subjects.

Sarcopenic patients had lower SBP, DBP, and MAP levels compared to the other study groups. In detail, SBP values were significantly lower in sarcopenic than in obese patients (*p* < 0.01) and lower than in controls (with borderline significance, *p *= 0.059).

DBP was lower in sarcopenic subjects than in the control group (*p* < 0.001), obese subjects (*p* < 0.001), and lower than SO patients (*p* < 0.01). Similarly, MAP was lower in the sarcopenic group than in the obese and control groups (*p* < 0.001 for both) and lower than the SO group (*p* < 0.05).

**Figure 1:**
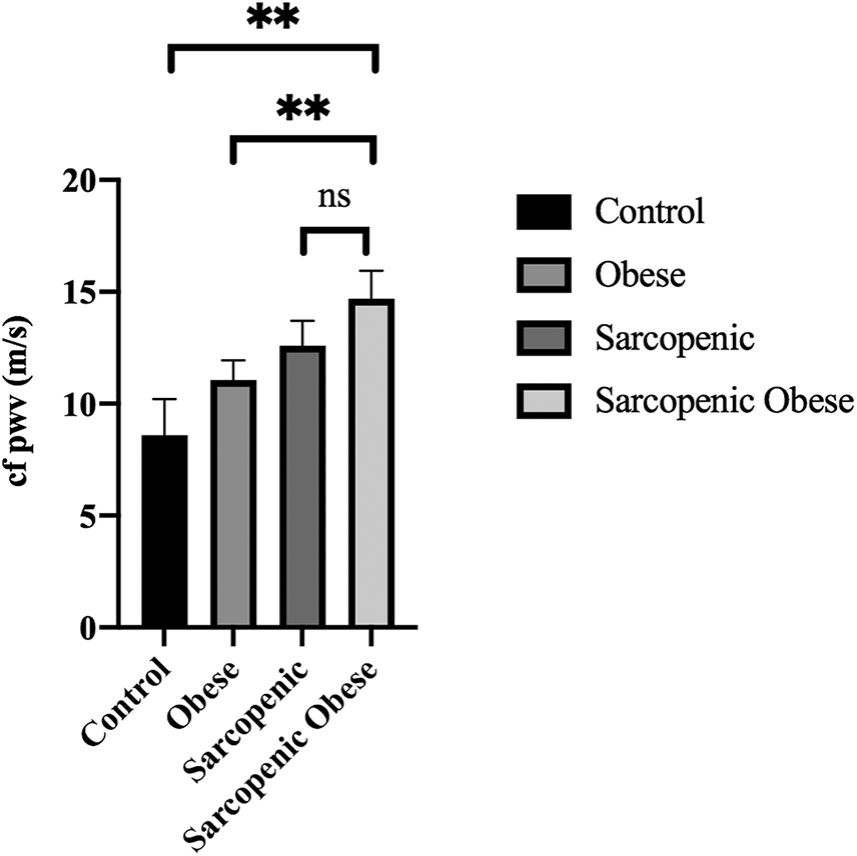
cfPWV values in different subgroups of the study population after adjustment for age, sex, MAP, and LDL cholesterol. (**p < 0.01).

CAVI has been found to be significantly higher in sarcopenic patients than in controls (11.31 ± 2.58 vs. 9.55 ± 1.77, *p* = 0.05) and obese group (11.31 ± 2.57 vs. 9.35 ± 1.97, *p* < 0.01), while cfPWV was higher in SO patients than in controls, obese, and sarcopenic subjects; after adjustment for age, sex, MAP, and LDL cholesterol, higher cfPWV was detected in SO compared to obese and controls (14.78 ± 1.27 m/s vs. 10.12 ± 1.23; *p* < 0.05 in controls, 10.84 ± 0.97; *p* < 0.05 in obese); although numerically higher in SO than in sarcopenic subjects, the latter comparison did not reach statistical significance (Figure 1).

In a multiple regression model (Table 3), considering cfPWV as a dependent variable and SO diagnosis, glycemia, heart rate, and MAP as independent variables, SO diagnosis resulted as a significant predictor of cfPWV.

**Table 3:** Multiple regression model considering cfPWV as an independent variable and sarcopenic obesity diagnosis, MAP, heart rate, and glycemia as dependent variables.

		Estimate	SE	t	*p*	*R* ^2^
cfPWV						0.20
	Constant	−8.13789	5.75658	−1.414	0.162	
	Sarcopenic Obesity	3.07544	1.39401	2.206	0.031	
	MAP	0.06057	0.04089	1.482	0.143	
	Glycemia	0.02695	0.01734	1.554	0.1246	
	Heart rate	0.11153	0.05219	2.137	0.036	

Furthermore, when considering CAVI as a dependent variable (Table 4) and sarcopenia diagnosis, glycemia, MAP, and heart rate as independent variables, sarcopenia was proven to be a significant predictor of CAVI. Even after adjustment for cfPWV, the association between sarcopenia and CAVI was confirmed, with a borderline significance (*p* = 0.059, not shown in the table).

**Table 4:** Multiple regression model considering CAVI as an independent variable and sarcopenia diagnosis, MAP, heart rate, and glycemia as dependent variables.

		Estimate	SE	t	*p*	*R* ^2^
CAVI						0.12
	Constant	11.244486	3.785603	2.970	0.004	
	Sarcopenia	1.378176	0.663873	2.076	0.042	
	MAP	−0.016030	0.027723	−0.578	0.565	
	Glycemia	0.011463	0.009248	1.239	0.219	
	Heart rate	−0.032911	0.027432	−1.200	0.234	

Even considering the separate role of muscle mass, fat mass and waist circumference on arterial stiffness indexes (Supplementary Table S1), multiple regression models showed that lower muscle mass (*p* = 0.01), along with height (*p* = 0.001), is associated with higher CAVI, whereas larger waist circumference (*p* = 0.009) along with lower muscle mass (*p* = 0.025) are significant predictors of cfPWV.

## Discussion

The main result of this cross-sectional study carried out on 77 older adults is that patients with sarcopenia and SO show increased arterial stiffness indices, that is, CAVI is higher in sarcopenic patients than controls and obese subjects, while cfPWV is higher in SO as compared to all the other groups. Noteworthily, sarcopenia and SO differ in terms of vascular involvement since SO seems to be more strongly associated with central aortic stiffness.

The physiological modifications of the cardiovascular system caused by aging and the prolonged exposure to several risk factors (33) are responsible for the increased arterial stiffness that has extensively been described in older adults, and which is confirmed by our results. Different segments of the arterial tree face different stiffening processes; in the setting of several diseases, a concomitant evaluation of both central and peripheral arterial segments, obtained by separate tools, provides a comprehensive overview of the stiffness status, opening the perspective on different pathophysiological conditions and on different clinical consequences.

Sarcopenia and SO are common conditions among older adults (11) and they represent interesting pathological models since they involve the peripheral muscular districts, yet presenting features of systemic diseases (34).

In our study, we evaluate both CAVI and cfPVW. CAVI provides an estimation of arterial stiffness from a broad proportion of the arterial tree, which includes both the aorta and the peripheral arteries. Peripheral stiffness is influenced by several factors; previous and consolidated evidence showed that the degree of arterial wall tethering increases toward the periphery (35), and it has been shown that PWV is not only affected by the elastic modulus of the arterial wall but also by the elastic modulus of the surrounding tissue (36). We observe higher CAVI in patients with sarcopenia, in line with a previous study by Kirkham and colleagues (37), who showed an independent association between sarcopenia and CAVI in 366 English subjects (aged over 45 years), and with another study published by Im et al. (38), who evaluated the association between arterial stiffness measured by CAVI and muscle mass deficit in 3,356 middle-aged Korean men. Actually, our study seems to add to these previous findings because it was performed in older populations and integrated measures of muscle strength into the body composition analyses.

A relationship between CAVI and sarcopenia has been recently found in a cohort of 100 older adults (aged 65 years and over), affected by heart failure (39).

Interestingly, providing a simultaneous measurement of CAVI and cfPWV, our study shows that while CAVI is negatively associated with SMI, no significant association was found between SMI and cfPWV, suggesting that central aortic stiffening is not primarily affected in the presence of sarcopenia (37).

Reflecting arterial stiffness from the aorta and peripheral arteries, CAVI is known to increase with aging, and it is higher in men and in several pathological conditions (30). In order to relieve the burden of central aortic stiffness, we included cfPWV in the regression model, eventually observing that sarcopenia diagnoses, which are derived from reduced muscle strength and reduced SMI, are associated with higher CAVI.

cfPWV is a proxy for central arterial stiffness and it is considered the gold standard technique by the European Guidelines on Hypertension (16); higher cfPWV is related to cardiovascular morbidity and mortality (15). However, in our study population, cfPWV is significantly higher in the SO group than in controls, obese, and sarcopenic subjects, and higher in SO group than in obese and control subjects even after adjustment for several variables. Moreover, SO diagnosis results as a significant predictor of cfPWV even in the regression model.

These results confirm and complement previous knowledge since we shed light on a novel predictor of cfPWV, namely, SO, besides the well-known association between central arterial stiffening and aging, male sex, and blood pressure (40).

Our results seem to demonstrate a synergic contribution of obesity and sarcopenia on vascular stiffness.

Previous studies suggested and demonstrated that visceral obesity and sarcopenia independently affect arterial stiffness (19); however, there is a lack of knowledge regarding central arterial stiffness in SO, and previous studies rely on baPWV (19), which is not a pure index of central stiffness since it encloses peripheral segments as well.

By obtaining cfPWV for each patient, we can enrich previous evidence, suggesting that in the presence of obesity, the whole arterial tree, including the central segments, is likely to present functional and structural damages. Thus, the increased central arterial stiffness, represented by higher cfPWV in our SO subgroup, may result from a heavier contribution of obesity (rather than sarcopenia) on vascular stiffness.

Robust evidence has widely shed light on the association between adipose tissue and vascular dysfunction (41, 42). Several mechanisms may explain the correlation between visceral fat and subclinical vascular damage. The abdominal adipose tissue is associated with increased levels of circulating fatty acids, which have a significant association with endothelial damage (43). Moreover, subjects with visceral adiposity have higher levels of IL-6, plasminogen activator inhibitor, TNF-alpha, and leptin, displaying a negative effect on the endothelium (44), and lower adiponectin, reducing the protective role of adiponectin itself on the endothelium (45). Thus, visceral obesity is considered to be responsible for proinflammatory cytokine release, resulting in altered muscle metabolism and activation of a catabolic vicious circle, eventually leading to the production of IL-6 and further degradation of skeletal muscle (46). According to this pathophysiological background, it is possible to hypothesize greater arterial damage in SO than in obese patients.

The strong correlation between arterial stiffness, SO, and sarcopenia observed in older adults in our study raises the question of whether vascular stiffness is a cause or consequence of sarcopenia. Our regression models demonstrate that SO diagnosis is a predictor of cfPWV, whereas low sarcopenia predicts a CAVI increase.

The vascular supply to muscle tissue is an intriguing feature. Previous evidence provided by the Health ABC study, conducted on 2,405 older adults, observed a reduction in baseline blood flow to the leg in the elderly, owing to an increased sympathetic vasoconstriction and arterial stiffness; thus, dysfunctional blood vessels dynamics, with a low level of chronic ischemia, could have an independent role on the genesis of sarcopenia (46).

On the other hand, sarcopenia may be interpreted as a cause of vascular impairment: the KNHANES study (12), conducted on 1,578 older Korean subjects, suggested that sarcopenia may be an independent risk factor for cardiovascular disease. An increase in fat mass and lipid infiltration, typically occurring in sarcopenia, with consequent macrophage-mediated release of proinflammatory cytokines and adipokines from adipocytes, resulting in chronic inflammation, may be involved in the link between sarcopenia and cardiovascular risk. In line with this hypothesis, Buford and colleagues previously underlined that in the presence of sarcopenia, harmful cytokines, oxidative stress, impaired hormone regulation, and cellular communication might impair vascular function and compliance (47).

The strengths and limitations of our study should be acknowledged in order to give a proper interpretation of the results. First, the present study provides a comprehensive evaluation of sarcopenia and SO. Second, we systematically provided a separate evaluation of central and peripheral arterial stiffness. Anyhow, our sample size was small and this may have reduced the statistical significance of some associations; it should be noted that muscle quality, and in particular myosteatosis, has not been investigated in our study, thus no conclusion should be made with respect to its role on arterial stiffness. Furthermore, we could not evaluate adipokines as well as insulin resistance, which could play a remarkable role in the relationship between SO and arterial stiffness.

The different trends in tonometric and CAVI-derived arterial stiffness should be carefully considered: central and peripheral arterial segments face different aging and stiffening processes due to a wide amount of anthropometric, structural, and physiological factors (48). When considering CAVI as an arterial stiffness index, it should be acknowledged that it is also affected by the perivascular tissue at the four limbs; therefore, a higher mass or density of the surrounding tissue and or a higher degree of tethering might dampen the propagation of the wave, resulting in a lower PWV (49). This may explain part of the association between sarcopenia and higher peripheral arterial stiffness.

Finally, our data, due to the cross-sectional nature of the study, do not allow us to draw any conclusion regarding a cause-effect relationship, and further research is needed to gain insight into the pathophysiological aspects; however, a reciprocal effect of impaired muscle quality and impaired vascular supply on each other cannot be excluded.

In conclusion, the results of our study demonstrate an association between sarcopenia and SO and arterial stiffness, among older subjects, describing increased arterial stiffness both in sarcopenic and SO patients. However, a distinction should be made. CAVI is significantly related to anthropometric parameters, namely, muscle mass and height, whereas greater central aortic stiffness (cfPWV) is described in SO, with a remarkable effect on cardiovascular risk. Given the widespread prevalence of sarcopenia and SO, especially among older adults, further insight is needed to examine the burden of this condition on the cardiovascular risk profile.

## Data Availability

The raw data supporting the conclusions of this article will be made available by the authors, without undue reservation.
