# Supplementary material for: Sarcopenia, sarcopenic obesity, and arterial stiffness among older adults
Source: Front Cardiovasc Med. 2024 Feb 8;11:1272854. doi: 10.3389/fcvm.2024.1272854 (PMC10885346; doi:10.3389/fcvm.2024.1272854)
Supplement: Supplementary file 1 [file Table1.docx]

**Sarcopenia, Sarcopenic Obesity and Arterial Stiffness among older adults**

**Supplementary Table 1**

| Dependent  variable |  | Independent  variables | Estimate | SE | t | p | R^2^ |
| --- | --- | --- | --- | --- | --- | --- | --- |
| CAVI |  |  |  |  |  |  | 0.29 |
|  |  | Constant | -3.51012 | 6.08616 | -0.577 | 0.566 |  |
|  |  | Waist Circumference | 0.01538 | 0.02765 | 0.556 | 0.580 |  |
|  |  | Muscle Mass | -0.10521 | 0.04063 | -2.589 | 0.012 |  |
|  |  | Fat Mass | -0.02663 | 0.02990 | -0.891 | 0.377 |  |
|  |  | Height | 0.12059 | 0.03530 | 3.417 | 0.001 |  |
|  |  | Heart Rate | -0.04947 | 0.02863 | -1.728 | 0.089 |  |
|  |  | cfPWV | 0.12707 | 0.05724 | 2.220 | 0.030 |  |
|  |  |  |  |  |  |  |  |
| cfPWV |  |  |  |  |  |  | 0.20 |
|  |  | Constant | -13.31119 | 13.09827 | -1.016 | 0.313 |  |
|  |  | Waist Circumference | 0.14908 | 0.05607 | 2.659 | 0.009 |  |
|  |  | Muscle Mass | -0.19777 | 0.08619 | -2.295 | 0.025 |  |
|  |  | Fat Mass | -0.05491 | 0.06388 | -0.859 | 0.393 |  |
|  |  | Heart Rate | 0.14462 | 0.06069 | 2.383 | 0.020 |  |
|  |  | Height | 0.06311 | 0.07668 | 0.823 | 0.414 |  |

Multiple regression models considering the separate role of muscle mass, fat mass and waist circumference on arterial stiffness indexes.
